# Motor control or graded activity exercises for chronic low back pain? A randomised controlled trial

**DOI:** 10.1186/1471-2474-9-65

**Published:** 2008-05-05

**Authors:** Luciana G Macedo, Jane Latimer, Chris G Maher, Paul W Hodges, Michael Nicholas, Lois Tonkin, James H McAuley, Ryan Stafford

**Affiliations:** 1 Musculoskeletal Division, The George Institute for International Health, 341 George St, Sydney, NSW 2000, Australia; 2 NHMRC Centre of Clinical Research Excellence in Spinal Pain, Injury and Health, School of Health and Rehabilitation Sciences, The University of Queensland, Brisbane, QLD 4072, Australia; 3 Pain Management & Research Centre, The University of Sydney at Royal North Shore Hospital, St Leonards, NSW 2065, Australia

## Abstract

**Background:**

Chronic low back pain remains a major health problem in Australia and around the world. Unfortunately the majority of treatments for this condition produce small effects because not all patients respond to each treatment. It appears that only 25–50% of patients respond to exercise. The two most popular types of exercise for low back pain are graded activity and motor control exercises. At present however, there are no guidelines to help clinicians select the best treatment for a patient. As a result, time and money are wasted on treatments which ultimately fail to help the patient.

**Methods:**

This paper describes the protocol of a randomised clinical trial comparing the effects of motor control exercises with a graded activity program in the treatment of chronic non specific low back pain. Further analysis will identify clinical features that may predict a patient's response to each treatment. One hundred and seventy two participants will be randomly allocated to receive either a program of motor control exercises or graded activity. Measures of outcome will be obtained at 2, 6 and 12 months after randomisation. The primary outcomes are: pain (average pain intensity over the last week) and function (patient-specific functional scale) at 2 and 6 months. Potential treatment effect modifiers will be measured at baseline.

**Discussion:**

This trial will not only evaluate which exercise approach is more effective in general for patients will chronic low back pain, but will also determine which exercise approach is best for an individual patient.

**Trial registration number:**

ACTRN12607000432415

## Background

### The problem of chronic low back pain

Low back pain is extremely costly causing great economic burden for Australia's health system, and considerable suffering for the individual [1]. Around 60–80% of the population will at some time exhibit low back pain [2-5] and of these 70 to 80% will have at least one recurrence [6]. Despite the enormous amount of resources directed to the treatment of chronic low back pain world wide, treatment for this health condition continues to have a low success rate [7-9]. The search for more effective ways to manage chronic low back pain is critical if we are to improve the health and quality of life for many Australians.

### Which exercise approach for chronic low back pain?

In Australia the most frequently used treatment for chronic low back pain is exercise [9]. Exercise, however, is not a single treatment. The types of exercise programs for chronic low back pain vary widely e.g. land-based exercise versus exercise in water, individual exercise versus group exercise, isolated trunk exercise versus whole body exercise. Unfortunately there is little or no evidence to help clinicians select the most effective type of exercise for an individual patient. This absence of evidence means that care is likely to be sub-optimal.

While some trials of exercise have reported large, durable and clinically important effects [10] others have not [11] Because the types of exercise programs for chronic low back pain vary widely [7] and patient presentations also vary widely it is unlikely that all programs are equally effective for all patients. Further, close examination of results of trials reveals that even in the positive trials not all the subjects had a good outcome. Consequently, a single summary statement on the effectiveness of 'exercise' for chronic low back pain would not be meaningful. This would be analogous to attempting a summary statement on the efficacy of 'drugs' for chronic low back pain, an approach that ignores the different classes and doses of drugs used in the treatment of low back pain.

Two very different approaches to exercise are motor control exercise [12] (sometimes called specific spinal stabilisation exercise) and a graded activity program [13] At present it is unclear which form of exercise is more effective in the management of chronic low back pain. In a literature review McGuirk [14] concluded that the motor control approach is the only exercise therapy that has been shown to achieve substantial and lasting reductions in pain. In contrast the 2004 European guideline [15] for management of chronic non-specific low back pain concludes: 'the use of a cognitive-behavioural approach, in which graded exercises are performed, using exercise quotas, appears to be advisable' (p 85). Despite this controversy, in New South Wales (NSW) WorkCover in Australia has decided that graded activity is the preferred approach and has commenced training each physiotherapist, chiropractor and osteopath (~2,300 practitioners) to implement this treatment. Within the physiotherapy profession this course of action has been questioned as not all believe that graded exercise is the preferred approach for all patients. There has been a considerable debate regarding the most appropriate form of supervised exercise for chronic low back pain, however, at present there is no evidence to inform this debate because there has never been a direct comparison of the two approaches.

Informed prescription of exercise requires evidence on the effectiveness of each type of exercise; and decision algorithms to assist clinicians to select the best form of exercise for an individual patient. This information is not currently available and clinical practice is likely to be suboptimal. The ability to identify patients with a greater chance of success has the potential to establish new knowledge, and save time and money associated with unsuccessful treatment. This is a unique goal of the proposed study.

### Predicting response to treatment

Most people with chronic low back pain are considered to have 'non-specific' low back pain, that is, they have no definitive structural diagnosis. It is a widespread clinical belief that it is possible to use clinical features to identify sub-populations of patients with non-specific low back pain who respond differently to treatment and there is some early evidence to support this view [16-18]. Recently Childs and colleagues [17] found that manipulative treatment resulted in a 50% reduction in pain for 44% of patients in their study. However a patient who was positive on their prediction rule had a 91% probability of a successful outcome with manipulation, while a patient who was negative had only a 7% probability of responding. At present there is no similar rule for exercise treatment of chronic low back pain. This is a major gap in knowledge. Not only will our proposed study determine which exercise approach is more effective in general for patients with chronic low back pain, but it will also determine which exercise approach is best for an individual patient. The identification of clinical features that predict response to treatment will save the enormous amounts of time and money that are currently wasted on unsuccessful exercise programs. With the large prevalence of chronic low back pain even modest predictive ability could make a substantial contribution to health systems worldwide.

## Methods and Discussion

### Overview of research design

The study will be a randomised controlled trial comparing graded activity and motor control exercise for patients with chronic low back pain. Each exercise program will consist of 12 individually supervised hour sessions over an eight-week period, 2 additional follow-up treatments at 4 and 10 months and a home exercise program. Outcomes will be measured at 2 months, 6 months and 12 months from baseline. Putative predictor variables will be taken from the baseline clinical assessments. The study design, procedures and informed consent were approved by The University of Sydney Human Research Ethics Committee.

### Hypothesis

i) That motor control exercise will be more effective in improving pain and disability at 2, 6 and 12 months.

ii) The effect of graded activity (versus motor control) is greater in patients who are deconditioned and have negative beliefs about their pain.

iii) The effect of motor control exercise (versus graded activity) is greater in patients with impaired control of movement and stability of the spine.

### Subject recruitment

A total of 172 subjects will be recruited from general practitioners, physiotherapy practices and public hospitals in Sydney and Brisbane. Contact has been made with these sites and consent letters have been obtained. Subjects will be screened to identify those who are unsuitable for exercise management of their low back pain because of significant co-morbidity such as serious spinal pathology or contraindication to exercise. To screen for serious pathology, the physiotherapist will conduct a diagnostic triage following the Royal College of General Practitioners' low back pain guidelines [19] and will be screened for contraindications to exercise as listed in the ACSM guidelines [20].

Subjects will be included if they meet all of the following **inclusion **criteria:

• Non-specific low back pain +/- leg pain persisting for at least 3 months duration

• Currently seeking care for low back pain

• Aged more than 18, less than 80 years

• English speaker

• Clinical assessment indicates that the subject is suitable for active exercises

• Expects to continue residing in the Sydney or Brisbane region for study duration

• Obtains a score of moderate or greater on question 7 or 8 of the SF-36.

(*Question 7 *– How much bodily pain have you had during the past week? None, very mild, mild, moderate, severe, very severe).

(*Question 8 *– During the past week, how much did pain interfere with your normal work, including both work outside the home and housework? Not at all, a little bit, moderately, quite a bit, extremely) [21].

Subjects will be **excluded **if they have any of the following:

• Known or suspected serious spinal pathology (fracture, metastatic, inflammatory or infective diseases of the spine, cauda equina syndrome/widespread neurological disorder).

• Nerve root compromise (at least 2 of the following signs: weakness/reflex changes/sensation loss, associated with the same spinal nerve)

• Previous spinal surgery or scheduled for major surgery during the treatment follow-up period

• Co-morbid health conditions that would prevent active participation in the exercise programs.

Specific spinal pathology or contraindication to treatment may be suspected based upon the results of the screening questionnaire and the Physical Activity Readiness Questionnaire. If the assessor suspects the presence of any pathology or contraindication to treatment, these subjects should be further investigated by a registered physiotherapist and medical clearance obtained if necessary.

Subjects receiving workers compensation will only be included in the trial if they have written consent from the nominated treating doctor, insurer and employer agreeing to their participation in the trial. The letters should state that they agree with the condition that only the trial treatments will be administered to the patient and acknowledges that the patient will be instructed not to seek other treatments during the trial treatment period.

### Assessment and Allocation

#### Randomisation

Participants will be allocated to treatment group using sealed opaque envelopes. Physiotherapists will allocate patients by drawing the next consecutive envelope allocated to their practice. A sticker containing the letter M (motor control) or G (graded activity) will be inside the envelope. After reading the sticker it will be attached to the patient's file. The therapist will not reveal the patient's allocation until the end of the study.

#### Outcome measures

Following the screening consultation, personal characteristics (age, gender, ethnicity, religion, weight, height. level of education, employment status, doctor's details and contact information) and information about symptoms of low back pain will be collected. The following treatment outcomes will be measured at baseline, 2 months, 6 months and 12 months.

##### Primary Outcome (at 2 and 6 months)

1. Average pain intensity over the last week (0–10 scale) [22,23]

2. Patient-generated measure of disability (Patient-Specific Functional Scale) [22,23]

##### Secondary Outcomes

3. Patient's global impression of change (Global Change) [22,23]

4. Condition-specific measure of disability (Roland Morris Disability Questionnaire) [24]

5. Generic measure of health status (SF-36 Version I) [21]

In addition subjects will be asked about their pain (average for preceding 24 hours and their average pain in the preceding week) once a month during one year. This data will be collected using a SMS (phone message) system. A SMS message will be sent monthly to all subjects reminding them to text the researcher with a number from 0–10 representing their average pain intensity over the preceding 24 hours and a number representing their average pain intensity over the preceding week. If subjects do not have a mobile phone they will be e-mailed monthly and asked to supply their average pain intensity score via return email. For patients that do not have a mobile phone or access to email, the average pain intensity will be recorded by phone. At the end of the study all participants will receive $10.00 to cover the cost of text messaging. A summary measure of the pain experienced over the 12-month period is provided by the area under the curve of the pain intensity/time curve. These measurements may better reflect the fluctuating nature of chronic pain than is possible with traditional outcome measures taken at a single time point. As the measures are novel the measurement properties are unclear and so we will regard this as a secondary outcome.

To maximise attendance at follow-ups subjects will receive a reminder letter and a phone call 24 hrs prior to their appointment. Every attempt (within ethical constraints) will be made to obtain outcome data, regardless of subject's compliance with trial protocols. Follow-up measures will be scored by an investigator who is blinded to group allocation. At 2 months, information about side effects of treatment will be collected from all subjects using open-ended questioning. At each follow-up, information on use of other pain-related treatments will be recorded as will the patient's compliance with their home exercise program.

### Treatment effect modifiers

The treatment rationale for the two types of exercise suggests potential treatment effect modifiers. The rationale for motor control exercise is that people with low back pain have poor control and coordination of the trunk muscles that results in control of movement and stability of the spine that is less than optimal [25,26]. This approach is based on a large body of literature that shows changes in muscle recruitment, movement and stability of the spine in patients with low back and pelvic pain. There is evidence from in-vivo and modelling studies that suggests that the muscle control strategies adopted in low back pain have the potential to prolong and prevent recovery from low back pain [25-27]. These deficits in control may vary from so called 'instability' to excessive stabilisation of the spine as a result of increased muscle activity. Motor control exercise uses a motor learning approach to re-establish normal control of the deep spinal muscles and then to coordinate the entire trunk muscle system during functional tasks. Logically this treatment is expected to work best in those who have impaired control of the spinal muscles, and in particular the deep muscles. *Accordingly we will take measures that reflect poor motor control of the spine e.g. trunk proprioception, trunk stiffness, trunk muscle response, tests of deep muscle control*. The rationale for graded activity is that people with chronic low back pain are generally deconditioned as a result of injury or disuse and that this deconditioning underpins their chronic symptoms. Additionally many people with chronic low back pain have beliefs about pain and injury that are unhelpful to recovery. With graded activity the physiotherapist teaches the patient how to increase their activity. Logically this treatment should work best in patients who are deconditioned and have unhelpful beliefs about their back pain. Patients with high levels of habitual activity are less likely to benefit. *Accordingly we will take measures of aerobic fitness, habitual activity level, kinesiophobia and self-efficacy*.

We will collect baseline data about clinical/demographic data, measures of beliefs and attitudes about pain, measures of physical activity and fitness and measures of control and coordination of the lumbar spine and pelvis.

#### Clinical/Demographic data

LBP screening questionnaire [28]

#### Measures of beliefs and attitudes about pain

PASS-20 (Pain Anxiety Symptom Scale) [29]

Pain Self Efficacy Questionnaire [30]

#### Measures of Physical activity and fitness

International Physical Activity Questionnaire (IPAQ) [31]

Three minute step test [32]

#### Measures of control and coordination of the lumbar spine and pelvis

##### Clinical lumbar spine instability test

As no in-vivo measures of "instability" have been validated assessment of instability is currently dependent on subjective data. A recent Delphi survey of international experts [33] has developed 15 questions where positive subject responses indicate increasing likelihood of lumbar spine instability. These questions include report of feelings of giving way, and frequent bouts or episodes of symptoms [33].

##### Test of trunk proprioception

Proprioception is a critical component of control and determines the effectiveness of control strategies. Although studies of repositioning have provided variable results [34,35], recent evidence suggests that people with low back pain have significant errors in sense of effort [36], which contributes to sense of position [37]. The contribution of sense of effort to proprioception will be measured using a standardised technique in which subjects are asked to generate forces aiming to achieve fluctuating targets. This test is performed first with and then without feedback of force. Studies have shown that individuals with LBP have difficulties in matching the forces in the direction of trunk extension [36].

##### Test of trunk stiffness

Active trunk stiffening by the trunk muscles can be evaluated in a dynamic task using an indirect measure of trunk stiffness and damping (Hodges et al, unpublished data). The response of the trunk to removal of a load is modelled as a second order system and resolved for stiffness, damping and mass. People with low back pain have been shown to have increased stiffness and decreased damping (Hodges et al, unpublished data)

##### Test of trunk muscle response

The response of the trunk muscles to removal of a load from the trunk has been shown to predict occurrence and recurrence of pain [38]. This measure evaluates the net response of the trunk muscles to a perturbation [39]. During a trunk perturbation test the onset and offset of the trunk flexors and extensors are measured using electromyography with surface electrodes over 12 muscles. People with LBP and those at risk for the development of low back pain have increased co-contraction of trunk flexors and extensors, and delayed offset of activity [38].

##### Test of deep muscle control

This test is a novel measure of a subject's ability to activate the deep abdominal muscles. Until recently recruitment of the deep abdominal muscles could only be assessed with invasive EMG recording techniques that have limited application in large clinical trials. We have recently solved this problem by developing an ultrasound test [25] that provides a non-invasive method to investigate the automatic recruitment of the trunk muscles in people with LBP in a clinical setting [25]. Both EMG and ultrasound measures have a good ability to discriminate patients with low back pain from pain-free controls [26].

We understand that the refined laboratory measures we propose may not all be viable in clinical practice. However it is important to use the best available measure to clearly establish the predictive ability of each factor. Equipped with this information we can then focus our attention on developing clinical versions of the tests that are shown to be important predictors.

### Interventions

Subjects in each group will receive 12 one-hour treatments over an 8 week period, i.e. 2 sessions/week in the first month and 1 session/week in the second month and 2 additional 1 hour follow-up sessions at 4 & 10 months. Patients will be advised to do home exercises for at least half an hour per week in the first month and one hour per week in the second month. However, the type of exercises, intensity and amount done per day will be performed at the discretion of the physiotherapists. The treatment session and home exercises will add up to 20 hours of treatment. In both exercise programs an individualised program for each patient is prescribed. The treatment sessions are designed to become less frequent over time to encourage independence. Patients with continued pain following all sessions will be asked to continue their home exercise program until pain-free for a week. If a recurrence occurs, re-initiation of the home program will be advised. This is consistent with current clinical practice.

• The *graded activity *program is based upon the treatment approach reported by Lindstrom [40] and identical to the protocol we used in a previous trial [13]. At the first visit the physical demands of subjects' work and home activities will be established and measures of mobility, strength and aerobic fitness taken. From these assessments an individualised, submaximal, progressively incremented exercise program will be developed to train those functions found to be inadequate for performance of work or leisure activities. The goal is to improve the subject's ability to complete functional activities they specify as being difficult to perform because of the low back pain. Principles underlying cognitive-behavioural therapy will be used by the physiotherapists in their training and supervision role. These principles include the encouragement of skill acquisition by modeling the exercises, providing information, setting progressively raised goals, self-monitoring of progress, and verbally reinforcing progress made by subjects towards their goals. Fear of increased pain or even possible (re)injury will be addressed by discussion of the realistic chances of exercises causing injury, by ensuring that subjects set initial goals well within their capabilities (to maximise the chances of early success experiences), and by encouraging subjects to maintain a graded increase in exercises and other activities with regular rest breaks and frequent reflection on achievements.

Each subject will carry out a form of aerobic exercise (e.g. walking or cycling), stretches, functional activities, activities to build speed, endurance and coordination and trunk and limb strengthening exercises. Subjects will be given an individualised home exercise program that will be regularly evaluated by the physiotherapist.

• The *motor control *exercise program is based upon the treatment approach reported by Richardson et al [27], O'Sullivan et al [10] and Moseley [41] and similar to the protocol we used in an earlier trial [12]. The approach utilises the principles of motor learning to retrain the optimal control and coordination of the lumbar spine and pelvis. Stage one involves regaining basic control strategies. This often involves training the pre-activation of the deeper muscles of the trunk such as transversus abdominis, multifidus, pelvic floor and diaphragm muscles that are typically affected in the presence of pain. The strategies that are selected are based on the patients presenting changes in coordination. Therapists will also assess and train posture and movement dysfunction (e.g. provocative movements and poor control strategies). Basic principles of motor learning such as simplification, segmentation, augmented feedback and practice are used to retrain the ideal activation of the deep muscles. Recent data suggests that these strategies restore control of the deep muscles [42] and lead to persistence of the change after cessation of treatment [43,44]. In this stage of training therapists will have access to a range of strategies that are typical in clinical practice including ultrasound imaging for feedback of muscle contraction, EMG biofeedback and other tools [27]. Other targets of this stage include coordination of breathing and changes at adjacent segments (e.g. hip, thoracic spine). In stage 2 participants will be progressed though to more complex static and dynamic tasks, and training of functional activities. At all progressions the therapist evaluates and corrects trunk muscle recruitment strategies, posture, movement patterns and breathing. Session 12 is a discharge session where the patient's progress will be reviewed and patients will be prescribed exercises to continue at home.

A treatment demarcation table was developed to help the trial clinicians to identify salient features from each of the interventions and to avoid unintended contamination between the two treatment approaches (see Table 1).

**Table 1:** Treatment Demarcation

**Principles**	**Graded Activity**	**Motor Control**
Goal setting	√	√
Pain contingent	X	√
Time contingent	√	X
Quotas/Pacing	√	X
Reinforce well behaviour and ignore illness behaviour	√	X
Education regarding pain system and reassurance	√	√
Education regarding ergonomic factors and body awareness	√	√
Generalised (whole body) exercises with consideration of specific muscle activity	X	√
Generalised (whole body) exercises without consideration of specific muscle activity	√	X
Specific (localised) exercises	X	√
Focus on correct activation of muscles	X	√
Correction of posture	X	√
Strength training	√	√
Cardiovascular/Fitness training	√	√
Coordination training	X	√
Correction of motor patterns	X	√
Muscle stretching	√	√
Breathing pattern	X	√
Consideration of continence	X	√
Correction of provocative movement patterns	X	√
Relaxation techniques	X	√
Progression to functional activities	√	√
Use feedback (e.g. US, EMG, biofeedback) to enhance learning of movement pattern or muscle activation.	X	√
Home exercises	√	√

Subjects in each treatment group will be asked not to seek other treatments for their chronic low back pain and where possible not to change current medications during the treatment period. Several mechanisms will be used to ensure that the trial protocol is consistently applied. Protocol manuals have been developed and staff will be trained to ensure that screening, assessment, randomisation and treatment procedures are conducted according to protocol. To ensure standardisation we will hold regular meetings with site visits. An independent researcher will monitor a randomly chosen subset to ensure adherence to assessment, randomisation and treatment procedures. Medical practitioners will be asked not to request the subject's allocation unless it is deemed necessary for medical care. At the completion of the exercise program, patients will be encouraged to continue the home exercise routine demonstrated at the discharge session. Subjects will be free to seek other treatment after the experimental period.

At 2 moths follow-up information about side-effects of treatment will be collected using open-ended questioning. Also at 2 months the compliance with the home exercises during the 8 weeks of treatment will be assessed. At 6 and 12 months open-ended questions about recurrence, recovery and about other treatment received for their low back pain during the study period will be sought. Reported recurrence can be compared against that recorded from the monthly SMS data.

### Data integrity

The integrity of trial data will be monitored by regularly scrutinising data sheets for omission and errors. Data will be double entered and the source of any inconsistencies will be explored and resolved.

### Data analysis

#### Treatment efficacy variables

In our primary analysis, we will use a regression model to test for the effect of treatment on outcome at 2, 6 and 12 months follow up. A treatment effect will be calculated for each of the follow-up time points and, if there is a statistically significant treatment effect at any time point, we will also calculate number needed to treat (NNT) to achieve pain recovery (pain ≤ 1 out of 10 (35)) and 95% confidence intervals.

##### Treatment effect modifiers

Individual variables will be tested for their association with treatment effect by adding a predictor × treatment group interaction term to the regression equation.

##### Sample size calculation

Brookes and colleague's [45] simulations demonstrate that trials have the same power to detect a treatment effect that is half the size of the interaction effect so sample size needs to be dictated by the size of the *interaction effect *that is to be detected. We argue that small interaction effects are not clinically significant and we have planned the study with sufficient power to detect the following clinically important interaction effects.

We have taken the SD estimates from our previous trials [12,13]. With specifications of alpha = 0.05, power = 0.80 and allowing for up to 10% loss to follow up and 10% treatment non-compliance a sample size of 86 participants per group will allow us to detect an interaction effect size of 1.0 SD (the smallest effect size we have specified above) and a treatment main effect of 0.5 SD (see Table 2). We understand the study is somewhat overpowered for the main treatment effect but it is crucial to adequately power the study for the interaction effect.

**Table 2:** Power calculations for main effects and interactions

Main effect	Interaction	Outcome (SD)
1.0	2.0	Pain intensity scale (SD = 2.0) [23, 48]
1	2	Patient Specific Functional Scale (SD = 1.8) [23, 48, 49]
2.5	5	Roland Morris Disability Questionnaire (SD = 4.9) [24]
1	2	Global Perceived Effects Scale (SD = 2.0) [22, 23]

### Justification of study design

#### Controlling bias

The trial includes key methodological features that have been recognised as minimising bias in clinical trials: true randomisation, concealed allocation, specification of eligibility criteria, blind outcome assessment, patient blinding, blind analysis and intention-to-treat analysis. The nature of the treatments precludes blinding of patient and treatment provider.

#### Predictor variables

We will use an exploratory approach and will test a wide range of potential predictors of treatment response. We acknowledge that any predictor identified in the analysis should be interpreted with caution until confirmed in subsequent independent studies.

#### Outcomes

Our choice of outcomes is consistent with best practice recommendations for clinical trials studying chronic pain [46] and back pain [47]. Measures of pain symptoms, disability and generic health status will be taken. We have supplemented the back-related disability measure (Roland-Morris) with a patient-generated measure of disability (Patient-Specific Functional Scale) because there is evidence that patient-generated measures of disability are more responsive than condition-specific measures [22,48,49].

## Conclusion

We have presented the rationale and design of a randomised controlled trial comparing the effect of motor control exercises and graded activity in a group of patients with chronic non specific low back pain with an analysis of treatment effect modifiers. The results of this trial will be published as soon as they are available.

## Competing interests

The authors declare that they have no competing interests.

## Authors' contributions

LGM, JL, CGM, PWH, MN, LT, JHM and RS were responsible for the design of the study. LGM and JHM will act as trial coordinators. All authors have read and approved the final manuscript.

## Pre-publication history

The pre-publication history for this paper can be accessed here:



## References

[B1] Bogduk N (2004). Management of chronic low back pain. MJA.

[B2] Waddell G (1987). A new clinical model for the treatment of low-back pain. Spine.

[B3] Hicks GE, Fritz JM, Delitto A, S.M. MG (2005). Preliminary development of a clinical prediction rule for determining which patients with low back pain will respond to a stabilization exercise program.. Arch Phys Med Rehab.

[B4] Kasai R (2006). Current trends in exercise management for chronic low back pain: comparison between strengthening exercise and spinal segmental stabilization exercise. J Phys Med Sci.

[B5] Freburger JK, Carey TS, Holmes GM (2006). Effectiveness of physical therapy for the management of chronic spine disorders: a propensity score approach. Phys Ther.

[B6] Goldby LJ, Moore AP, Doust J, Trew ME (2006). A randomized controlled trial investigating the efficiency of musculoskeletal physiotherapy on chronic low back disorder. Spine.

[B7] Hayden JA, van Tulder MW, Malmivaara A, Koes BW (2005). Exercise therapy for treatment of non-specific low back pain. Cochrane Database Syst Rev.

[B8] Waddell G, Livingstone C (1998). The clinical course of low back pain. The Back Pain Revolution.

[B9] Walker BF, Muller R, Grant WD (2004). Low back pain in Australian adults: health provider utilization and care seeking. J Manipulative Physiol Ther.

[B10] O'Sullivan PB, Phyty GD, Twomey LT (1997). Evaluation of specific stabilizing exercise in the treatment of chronic low back pain with radiologic diagnosis of spondylolysis or spondylolisthesis.. Spine.

[B11] Yelland MJ, Glasziu PP, Bogduck N, Schluter PJ, McKernon M (2004). Prolotherapy injections, saline injections and exercises for chronic low back pain: a randomized trial. Spine.

[B12] Ferreira ML, Ferreira PH, Latimer J, Herbert RD, Hodges PW, Jennings MD, Maher CG, Refshauge KM (2007). Comparison of general exercise, motor control exercise and spinal manipulative therapy for chronic low back pain: A randomized trial. Pain.

[B13] Pengel LH, Refshauge KM, Maher CG, Nicholas MK, Herbert RD, McNair P (2007). Physiotherapist-directed exercise, advice, or both for subacute low back pain: a randomized trial. Annals of Internal Medicine.

[B14] McGuirk B, King W, Govind J, Lowry J, Bogduk N (2001). Safety, efficacy, and cost effectiveness of evidence-based guidelines for the management of acute low back pain in primary care. Spine.

[B15] Cost B13 working group (2004). European Guidelines for the Management of Chronic Non-Specific Low Back Pain. Cost B13 working group on guidelines for chronic low back pain.

[B16] Brennan GP, Fritz JM, Hunter SJ, Thackeray A, Delitto A, Erhard RE (2006). Identifying subgroups of patients with acute/subacute "nonspecific" low back pain: results of a randomized clinical trial. Spine.

[B17] Childs JD, Fritz JM, Flynn TW, Irrgang JJ, Johnson KK, Majkowski GR, Delitto A (2004). A clinical prediction rule to identify patients with low back pain most likely to benefit from spinal manipulation: a validation study. Annals of Internal Medicine.

[B18] Fritz JM, Brennan GP, Clifford SN, Hunter SJ, Thackeray A (2006). An examination of the reliability of a classification algorithm for subgrouping patients with low back pain. Spine.

[B19] Waddell G, Feder G, McIntosh A, Lewis M, Hutchison A, Practitioners RCG (1996). Low back pain evidence review.

[B20] ACSM ACSM's Guidelines for Exercise Testing and Prescription.

[B21] Sanson-Fisher RW, Perkins JJ (1998). Adaptation and validation of the SF-36 Health Survey for use in Australia. J Clin Epidemiol.

[B22] Pengel LH, Refshauge KM, Maher CG (2004). Responsiveness of pain, disability, and physical impairment outcomes in patients with low back pain.[see comment]. Spine.

[B23] Scrimshaw SV, Maher CG (2001). Responsiveness of visual analogue and McGill pain scale measures. J Manipulative Physiol Ther.

[B24] Roland M, Morris R (1983). A study of the natural history of back pain. Part I: development of a reliable and sensitive measure of disability in low-back pain. Spine.

[B25] Ferreira PH, Ferreira ML, Hodges PW (2004). Changes in recruitment of the abdominal muscles in people with low back pain: ultrasound measurement of muscle activity. Spine.

[B26] Hodges PW, Richardson CA (1996). Inefficient muscular stabilization of the lumbar spine associated with low back pain. A motor control evaluation of transversus abdominis. Spine.

[B27] Richardson CA, Jull GA, Hodges PW (1999). Therapeutic Exercise for Spinal Segmental Stabilization in Low Back Pain.

[B28] Kendall N, Linton S, Main C (1997). Guide to Assessing Psychosocial Yellow Flags in Acute Low Back Pain: Risk Factors for Long Term Disability and Work Lossed. Accident Rehabilitation and Compensation Insurance Corporation of New Zealand and the National Health Committee.

[B29] McCraken LM, Dhingra L (2002). A short version of the pain anxiety symptoms scale (PASS-20): preliminary development and validity. Pain Res Manage.

[B30] Nicholas MK (2007). Pain self-efficacy questionnaire: taking pain into account. Eur J Pain.

[B31] Craig CL, Marshall AL, Sjostrom M, Bauman AE, Booth ML, Ainsworth BE, Pratt M, Ekelund U, Yngve A, Sallis JF, Oja P (2003). International physical activity questionnaire: 12-country reliability and validity. Med Sci Sports Exerc.

[B32] Singh SJ, Morgan MDL, Scott S, Walters D, Hardman AE (1992). Developments of a shuttle walking test of disability in patients with chronic airways obstruction. Thorax.

[B33] Cook C, Brismee JM, Sizer PS (2006). Subjective and objective descriptors of clinical lumbar spine instability: a Delphi study. Manual Therapy.

[B34] Gill KP, Callaghan MJ (1998). The measurement of lumbar proprioception in individuals with and without low back pain. Spine.

[B35] Newcomer KL, Laskowski ER, Yu B, Johnson JC, An KN (2000). Differences in repositioning error among patients with low back pain compared with control subjects. Spine.

[B36] Coppieters M, Markussen H, Ajja R, Herland K, Selbaek H, Ansersen J, Hodges PW (2007). Task accuracy is reduced in patients with low back pain if visual feedback about task performance is removed.

[B37] Gandevia SC, Smith LJ, Crawford M, Proske U, Taylor J (2006). Motor control commands contribute to human position sense. J Physiol (Lond).

[B38] Cholewicki J, Silfies SP, Shah RA, Greene HS, Reeves NP, Alvi K, Goldberg B (2005). Delayed trunk muscle reflex responses increase the risk of low back injuries. Spine.

[B39] Radebold A, Cholewicki J, Panjabi MM, Patel TC (2000). Muscle response pattern to sudden trunk loading in healthy individuals and in patients with chronic low back pain. Spine.

[B40] Lindstrom I, Ohlund C, Eek C, Wallin L, Peterson LE, Fordyce WE, Nachemson AL (1992). The effect of graded activity on patients with subacute low back pain: a randomized prospective clinical study with an operant-conditioning behavioral approach. Physical Therapy.

[B41] Moseley L (2002). Combined physiotherapy and education is efficacious for chronic low back pain. Australian Journal of Physiotherapy.

[B42] Tsao H, Hodges PW (2007). Immediate changes in feedforward postural adjustments following voluntaty motor training. Exp Brain Res.

[B43] Tsao H, Hodges PW (2007). Persistence of improvement in postural strategies following motor control traning in people with recurrent low back pain. J Electromyogr Kisnesiol.

[B44] Tsao H, Hodges PW (2005). Specific abdominal retraining alters motor co-ordination in people with persistent low back pain.

[B45] Brookes ST, Whitley E, Peters TJ, al. (2001). Subgroup analyses in randomised controlled trials: quantifying the risks of false-positives and false-negatives. Health Technol Assess.

[B46] Dworkin RH, Turk DC, Farrar JT, Haythornthwaite JA, Jensen MP, Katz NP, Kerns RD, Stucki G, Allen RR, Bellamy N, Carr DB, Chandler J, Cowan P, Dionne R, Galer BS, Hertz S, Jadad AR, Kramer LD, Manning DC, Martin S, McCormick CG, McDermott MP, McGrath P, Quessy S, Rappaport BA, Robbins W, Robinson JP, Rothman M, Royal MA, Simon L, Stauffer JW, Stein W, Tollett J, Wernicke J, Witter J (2005). Core outcome measures for chronic pain clinical trials: IMMPACT recommendations. Pain.

[B47] Deyo RA, Battie M, Beurskens AJ, Bombardier C, Croft P, Koes B, Malmivaara A, Roland M, Von Korff M, Waddell G (1998). Outcome measures for low back pain research. A proposal for standardized use. Spine.

[B48] Maher C, Latimer J, Refshauge K (2001). Atlas of clinical tests and measures for low back pained: Melbourne..

[B49] Stratford P, Gill C, Westaway M, Binkley J (1995). Assessing disability and change on individual patients: a report of  a  patient specific measure. Physiother Can.

